# Influence of PSA level at salvage radiotherapy on metastasis-free survival following radical prostatectomy

**DOI:** 10.1007/s00345-025-05840-w

**Published:** 2025-11-21

**Authors:** Mike Wenzel, Benedikt Lauer, Kathrin Burdenski, Nikolaos Tselis, Claus Rödel, Christian Brandts, Marit Ahrens, Jens Köllermann, Markus Graefen, Clara Humke, Carolin Siech, Benedikt Hoeh, Felix K. H. Chun, Philipp Mandel

**Affiliations:** 1 Department of Urology, Goethe University Hospital Frankfurt, University Hospital Frankfurt, Goethe University Frankfurt am Main, Frankfurt am Main, Germany; 2 University Cancer Center Frankfurt (UCT), Frankfurt am Main, Germany; 3 Department of Radiation Oncology, University Hospital Frankfurt, Goethe University Frankfurt am Main, Frankfurt, Germany; 4 Department of Hematology/Oncology, University Hospital Frankfurt, Goethe University Frankfurt am Main, Frankfurt, Germany; 5 Department of Pathology, University Hospital Frankfurt, Goethe University Frankfurt am Main, Frankfurt, Germany; 6 Martini-Klinik Prostate Cancer Center, University Hospital Hamburg-Eppendorf, Hamburg, Germany

**Keywords:** RARP, Adjuvant, Salvage, Radiotherapy, Prostate cancer

## Abstract

**Purpose:**

The optimal time point of salvage radiotherapy (sRT) for biochemical recurrence (BCR) following radical prostatectomy is still under debate. Current European guidelines recommend salvage intensity-modulated and image‐guided radiotherapy for men with two consecutive PSA rises. However, no specific PSA threshold for initiation time is recommended. Nonetheless, lower PSA level may be associated with better cancer-control outcomes.

**Methods:**

Relying on the University Cancer Center Frankfurt database, we evaluated differences in metastasis-free survival (MFS) among patients treated with early sRT (< 0.5 ng/ml) vs. sRT at PSA ≥ 0.5ng/ml. Subgroup analyses addressed sRT patients with high-risk features for indication of adjuvant radiation therapy, including Gleason score 8–10 and/or pT3–4 stage and/or pN1.

**Results:**

Of 190 sRT patients, 69% received early sRT at median PSA 0.24ng/ml vs. 0.89ng/ml. MFS was significantly better for early sRT patients, relative to sRT at PSA > 0.5 ng/ml (hazard ratio [HR]: 8.44, *p* < 0.01). Similarly, sRT patients with high-risk features also had significant better MFS at sRT with PSA < 0.5ng/ml (HR: 12.69, *p* < 0.01). After additional multivariable adjustment, early sRT at PSA < 0.5ng/ml was independently associated with better MFS outcomes for all patients (HR: 8.2) and high-risk sRT subgroups (HR: 55.6, both *p* < 0.05). Finally, we validated the initiation of sRT at an even lower cut-off of a PSA level ≤ 0.25ng/ml. However, this did not result in a significantly different outcomes, probably due to sample size limitations.

**Conclusion:**

Our results validate European guidelines’ recommendation to initiate sRT at low PSA levels < 0.5ng/ml providing better MFS, especially in patients with high-risk features, refusing adjuvant radiation therapy.

**Supplementary Information:**

The online version contains supplementary material available at 10.1007/s00345-025-05840-w.

## Introduction

Adjuvant radiotherapy (aRT), which is administered before the onset of biochemical recurrence, is mainly indicated in patients with high-risk pathological features such as Gleason score 8–10, pT3–4, positive surgical margins or pN1 disease [1]. The efficacy of aRT has also been demonstrated in numerous studies, however, it is refused by some patients due to possible side effects and toxicity [2–5]. According to the guidelines of the European Urology Association (EAU), intensity-modulated and image-guided salvage radiotherapy (sRT) is recommended in cases of biochemical recurrence with two consecutive PSA elevations after radical prostatectomy. A specific PSA threshold for SRT initiation is currently not recommended [1]. Risk factors such as the PSA doubling time, pathological Gleason score, pathological T- and N-stage or positive surgical margins play a decisive role in this context [1]. Studies have shown that sRT significantly improves oncological outcomes, measured in terms of biochemical recurrence-free survival (BCR), metastasis-free survival (MFS) and overall survival (OS), relative to observation [6–8].

Comparative analyses of sRT vs. aRT demonstrated no significant differences in oncological outcomes when achieving equivalent results within short- and mid-term follow-up period [9–12]. Nevertheless, current guidelines and several studies recommend aRT for patients with adverse pathological features (e.g., pT3–4, Gleason score 8–10, or positive surgical margins) to reduce the risk of recurrence [1, 5, 13]. In that contextcurrent studies suggest that sRT may be administered as early as possible [14, 15]. Moreover, recently reported data from Tilki et al. in 2023 suggested sRT is even more effective when initiated at a PSA level < 0.25 ng/ml [15]. This hypothesis still requires further validation.

We addressed this void by relying on data of the University Cancer Center Frankfurt (UCT) database and conducted the current study to evaluate the oncological outcomes of sRT patients after radical prostatectomy addressing MFS. We assessed both the timing of sRT initiation and its efficacy especially in subgroups of high-risk patients with pT3–4 and/or Gleason score 8–10, and/or pN1 stage. We hypothesized that sRT and its cancer-control outcome may be directly associated with PSA level at initiation, especially in patients with high-risk features after radical prostatectomy.

## Materials and methods

### Study population

Patient data were provided by the University Cancer Center Frankfurt (UCT) after obtaining approval from the local ethics committee (reference number: SUG-4-2024). Adhering to the principles of the Declaration of Helsinki, we conducted a retrospective analysis of all prostate cancer patients treated with radical prostatectomy and subsequent sRT with a curative intent at the Department of Urology or the Department of Radiation Oncology at University Hospital Frankfurt, Germany, between 2014 and 2024. The study included sRT patients with BCR following radical prostatectomy. BCR was defined as two consecutive PSA rises following radical prostatectomy, or according to older definitions such as PSA level above ≥ 0.2 ng/ml [1]. Patients with metastatic disease at diagnosis/ prior to surgery were excluded. Based on these criteria, a total of 190 patients with BCR after radical prostatectomy and subsequent sRT were included in the study. All treatment decisions were made following individuals’ case discussions in a multidisciplinary tumor board.

### Definition of sRT

SRT was defined as radiation therapy to the prostatic bed (+/- pelvic lymph nodes) after occurrence of BCR in patients without PSA persistence, at least six months after radical prostatectomy, as described in EAU guidelines [1]. SRT was delivered in doses in accordance with the EAU/ESTRO guidelines at time point of initiation [1]. SRT was administered with concomitant ADT on physicians’ choice.

### Statistical analysis

Descriptive statistics involved calculating the frequencies and proportions of the categorical variables included in the analysis. For continuous variables, median values and interquartile ranges (IQR) were reported. The Chi-square test was used to evaluate the statistical significance of differences in proportions, while the t-test and Kruskal-Wallis test were applied to analyze differences in distributions. Continuous variables, including medians and interquartile ranges (IQR) were determined.

Primary study endpoint was MFS, defined as the time from radical prostatectomy to the first occurrence of clinically or radiologically confirmed distant metastasis. MFS outcomes were evaluated by using Kaplan-Meier curve analyses and log-rank tests in an early sRT group with PSA < 0.5 ng/ml vs. ≥0.5 ng/ml. Subsequently, a stricter cut-off of PSA ≤ 0.25 ng/ml, as suggested by Tilki et al., was validated using the same analytical approach/analyses [15]. Subgroup analyses for both PSA level stratifications were made for high-risk patients, defined as pT3–4, Gleason score 8–10 and/or pN1 status. All of the above analyses were further repeated in MFS landmark analyses starting follow-up period from time of sRT.

For all analyses, univariable and multivariable Cox regression models were applied. Multivariable models were adjusted to maximally account for potential baseline confounders, including patient-related factors (age, Eastern Cooperative Oncology Group [ECOG] performance status) and tumor-related characteristics (pT-stage, pN-stage, Gleason score, positive surgical margins). All statistical analyses and graphical representations were performed using the R software environment for statistical computing and graphics (version 3.4.3).

## Results

### Descriptive analyses

The total study population consisted of 190 sRT patients with a median age of 65 years at sRT (IQR 60–70 years, Table 1). ECOG performance status was 0 in 93% of all patients. At initiation of sRT, median PSA level was 0.33 ng/ml (IQR 0.20–0.61 ng/ml). Prior to radical prostatectomy, 77% of patients were classified as D’Amico high-risk category. Additionally, a total of 116 patients exhibited high-risk features such as pT3–4, and/or Gleason score 8–10 and/or pN1 stage.

**Table 1:** Descriptive baseline characteristics of 190 patients treated with sRT at a PSA level <0.5 ng/ml vs. ≥0.5 ng/ml

Characteristic	*N*	Overall *N* = 190	sRT at PSA <0.5 ng/ml *N* = 132 (69%)^a^	sRT at PSA ≥0.5 ng/ml *N* = 58 (31%)^a^	*p*-value^b^
Median age at sRT (yr)	190	65 (60, 70)	64 (64, 68)	68 (61, 71)	0.029
Highest PSA (ng/ml)	158	9 (6, 15)	9 (6, 14)	11 (7, 20)	0.10
PSA nadir (ng/ml)	190	0.17 (0.03, 038)	0.12 (0,02, 0.25)	0.66 (0.14, 1.24)	<0.001
PSA prior sRT (ng/ml)	190	0.33 (0.20, 0.61)	0.24 (0.17, 0.34)	0.89 (0.65, 2.00)	<0.001
BMI	78	26.1 (24.2, 27.7)	26.1 (23.9, 28.7)	26.0 (24.2, 26.9)	0.60
Amount of biopsy cores	69	12 (12, 14)	12 (12, 14)	12 (11, 14)	0.40
Positive biopsy cores	68	5 (4, 8)	5 (4, 8)	6 (4, 9)	0.30
ECOG status					
0	169	158 (93%)	111 (96%)	47 (89%)	0.10
1		11 (6.5%)	5 (4.3%)	6 (11%)	
Gleason ≥8	189	45 (24%)	27 (20.9%)	18 (30.6%)	0.30
Risk stratification after d’Amcio					
Low-risk	190	2 (1.1%)	2 (1.5%)	0 (0%)	>0.90
Intermediate-risk		41 (22%)	29 (22%)	12 (21%)	
High-risk		147 (77%)	101 (77%)	46 (79%)	
cT status					
cT1-2	188	82 (44%)	63 (48%)	19 (33%)	0.045
cT3-4		106 (57%)	67 (52%)	39 (67%)	
pT status					
pT1-2	187	81 (43%)	62 (48%)	19 (33%)	0.0051
pT3-4		106 (57%)	67 (52%)	39 (67%)	
pN status					
pN0	190	159 (84%)	114 (86%)	45 (78%)	0.082
pN1		15 (7.9%)	11 (8.3%)	4 (6.9%)	
pNx		16 (8.4%)	7 (5.3%)	9 (16%)	
PSM					
R0	190	142 (75%)	96 (73%)	46 (79%)	0.80
R1		44 (23%)	33 (25%)	11 (19%)	
Rx		4 (2.1%)	3 (2.3%)	1 (1.7%)	
Stadium					
I	189	8 (4.2%)	6 (4.6%)	2 (3.4%)	0.30
II		71 (38%)	54 (41%)	17 (29%)	
III		95 (50%)	60 (46%)	35 (60%)	
IV		15 (7.9%)	11 (8.4%)	4 (6.9%)	

*sRT* salvage radiation therapy; *ECOG* Eastern Cooperative Oncology Group; *IQR* interquartile range; *PSM* positive surgical margins

^a^Data are presented as median (IQR) or n (%)

^b^Kruskal-Wallis rank sum test; Fisher’s exact test; Pearson’s Chi-square test

**Table 2:** Descriptive baseline characteristics of 116 patients with high-risk features treated with sRT at a PSA level <0.5 ng/ml vs. ≥0.5 ng/ml

Characteristic	*N*	Overall *N* = 116	sRT at PSA <0.5 ng/ml *N* = 75 (69%)^a^	sRT at PSA ≥0.5 ng/ml *N* = 41 (35%)^a^	*p*-value^b^
Median age at sRT (yr)	116	66 (61, 70)	65 (61, 69)	67 (61, 71)	0.4
Highest PSA (ng/ml)	98	10 (7, 20)	9 (7, 15)	14 (8, 22)	0.026
PSA nadir (ng/ml)	116	0.19 (0.03, 0.50)	0.11 (0.02, 0.24)	0.79 (0.27, 1.30)	<0.001
PSA prior sRT (ng/ml)	116	0.37 (0.22, 0.69)	0.25 (0.17, 0.37)	0.92 (0.66, 2.00)	<0.001
BMI	51	26.42 (24.59, 27.15)	26.42 (24.84, 28.73)	26.42 (24.54, 26.85)	0.50
Amount of biopsy cores	45	12 (12, 13)	12 (12, 13)	12 (11, 18)	0.90
Positive biopsy cores	44	7 (4, 9)	6 (4, 9)	7 (5, 10)	0.30
ECOG status					
0	106	96 (91%)	62 (93%)	34 (87%)	0.50
1		10 (9.4%)	5 (7.5%)	5 (13%)	
Gleason ≥8	116	45 (38%)	27 (36%)	18 (44%)	0.40
Risk stratification after d’Amcio					
Low-risk	116	0 (0%)	0 (0%)	0 (0%)	>0.90
Intermediate-risk		1 (0.9%)	1 (1.3%)	0 (0%)	
High-risk		115 (99%)	74 (99%)	41 (100%)	
cT status					
cT1-2	115	9 (7.8%)	7 (9.5%)	2 (4.9%)	0.30
cT3-4		106 (92%)	67 (91%)	39 (95%)	
pT status					
pT1-2	115	9 (7.8%)	7 (9.5%)	2 (4.9%)	0.50
pT3-4		106 (92%)	67 (91%)	39 (95%)	
pN status					
pN0	116	93 (80%)	61 (81%)	32 (78%)	0.20
pN1		15 (13%)	11 (15%)	4 (9.8%)	
pNx		8 (6.9%)	3 (4.0%)	5 (12%)	
PSM					
R0	116	81 (70%)	48 (64%)	33 (80%)	0.13
R1		32 (28%)	25 (33%)	7 (17%)	
Rx		3 (2.6%)	2 (2.7%)	1 (2.4%)	
Stadium					
I	116	1 (0.9%)	1 (1.3%)	0 (0%)	0.80
II		5 (4.3%)	3 (4.0%)	2 (4.9%)	
III		95 (82%)	60 (80%)	35 (85%)	
IV		15 (13%)	11 (15%)	4 (9.8%)	

*ECOG* Eastern Cooperative Oncology Group; *IQR* interquartile range; *PSM* positive surgical margins; *sRT* salvage radiation therapy

^a^Data are presented as median (IQR) or n (%)

^b^Kruskal-Wallis rank sum test; Fisher’s exact test; Pearson’s Chi-square test

### Baseline characteristics early sRT: PSA < 0.5 ng/ml vs. ≥0.5 ng/ml

A total of 69% (*n* = 132) of patients received early sRT (PSA < 0.5 ng/ml), while 31% (*n* = 58) underwent sRT at a PSA level ≥ 0.5 ng/ml (Table 1). Median PSA in the early sRT cohort was 0.24 ng/ml (IQR 0.17–0.34 ng/ml) vs. 0.89 ng/ml (IQR 0.65–2.00 ng/ml) in the standard sRT group. Median age was significantly younger in the early sRT group (64 vs. 68 years, *p* = 0.03). No difference in ECOG performance status 0 was observed between both groups (96% vs. 89%, *p* = 0.1). Despite significantly lower tumor infiltration in biopsy cores in the early sRT group (60% vs. 80%, *p* = 0.042), no further statistically significant or clinically relevant differences were observed for pathological features, such as positive surgical margins (25% vs., 19%), pathological Gleason score 8–10 distribution (21% vs. 31%) or pN1 rates (8.3% vs. 6.9%; all *p* ≥ 0.08).

Of 116 sRT patients with high-risk features, baseline patient and tumor characteristics distributions were largely comparable between early vs. standard sRT patients, despite PSA prior to sRT (0.25 vs. 0.92 ng/ml, *p* < 0.001) (Table 2).

### MFS in early sRT: PSA < 0.5 vs. ≥0.5 ng/ml

**Fig. 1:**
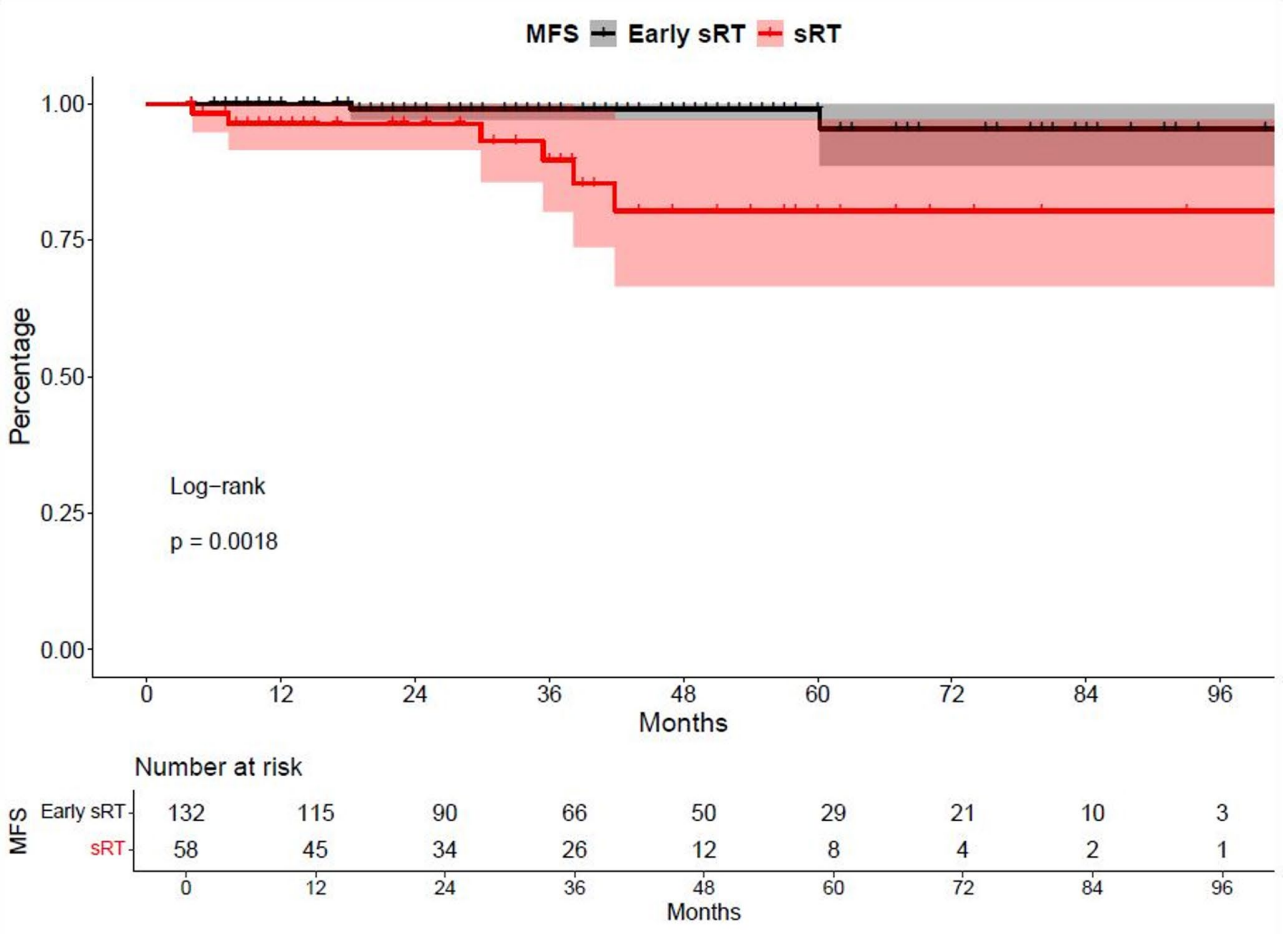
Kaplan-Meier curves depicting metastasis-free survival (MFS) of patients receiving salvage radiation therapy (sRT) at a PSA-level of < 0.5 ng/ml vs. ≥0.5 ng/ml

In MFS analyses, significant differences were observed between early sRT with PSA < 0.5 ng/ml vs. sRT at a PSA level ≥ 0.5 ng/ml (hazard ratio [HR]: 8.44, *p* < 0.01, Fig. 1) with median MFS not reached for both groups. Rates of 24- and 48-months MFS were 99% vs. 96.4% and 99% vs. 80.4%, respectively for early sRT vs. sRT. After multivariable adjustment in Cox regression models, the sRT group with PSA level ≥ 0.5 ng/ml was independently at higher risk of metastasis (HR: 8.2, *p* = 0.019, Supplementary Table 3).

**Fig. 2:**
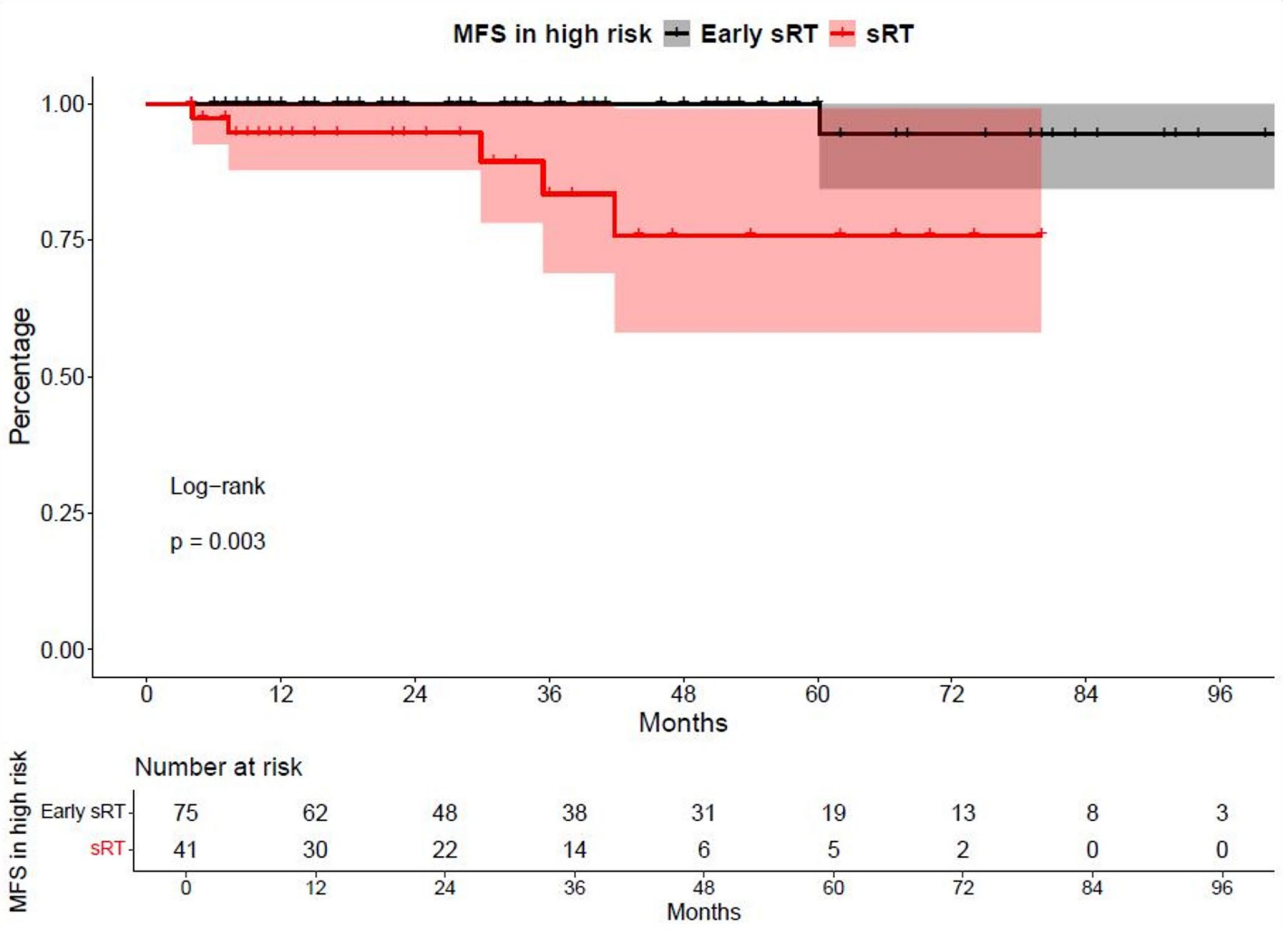
Kaplan-Meier curves depicting metastasis-free survival (MFS) of patients with high-risk features receiving salvage radiation therapy (sRT) at a PSA-level of < 0.5 ng/ml vs. ≥0.5 ng/ml

In subgroup analyses of sRT patients with high-risk features (Fig. 2), the early sRT group (PSA < 0.5 ng/ml) also demonstrated significantly better MFS outcomes, compared to the sRT group with PSA > 0.5 ng/ml (HR: 12.69, *p* < 0.01). Rates of 24- and 48-months MFS were 100% vs. 94.7% and 100% vs. 75.9%, respectively for early sRT vs. sRT with PSA > 0.5 ng/ml. In multivariable adjusted Cox regression models, the sRT group with PSA level ≥ 0.5 ng/ml was associated with a significantly higher risk of metastases (HR: 55.6, *p* = 0.029, Supplementary Table 4). Landmark analyses from time point of sRT initiation revealed similar significant results for overall and high-risk analyses.

Finally, we validated the initiation of sRT at an even lower cut-off of a PSA level ≤ 0.25ng/ml with virtually similar results.

## Discussion

We initially hypothesized that initiating sRT at lower PSA level may be associated with improved cancer-control outcomes such as MFS. To test these hypotheses, we examined MFS outcomes in patients treated at PSA levels < 0.5 ng/ml, and also tested this cut-off in sRT patients with high-risk features such as positive surgical margins, pathological Gleason score 8–10 and/or pN1 rates. We relied on data from the University Cancer Center Frankfurt and made several important observations.

First, when examining baseline characteristics of our total population, we observed a median age of 65 years, which is consistent with findings from previous studies [16, 17]. For example, Tilki et al. reported a comparable median age of 64 years in their overall study population of *n* = 25.551 sRT patients, treated between 1990 and 2020 [11, 15]. Similarly, a multicenter study by Parker et al. evaluating the timing of radiotherapy in a cohort of 1.396 patients, reported a median age of 65 years [11]. Conversely, a study by Stephenson et al. with the purpose of predicting the outcome of sRT showed a slightly younger median age of the population of 62 years. This may be caused by the fact that they measured the age pre prostatectomy [18]. Moreover, a significantly younger age in the early sRT group compared to the standard sRT group was detected in our study (64 vs. 68 years, *p* = 0.029), which may be related to a stricter follow-up in younger prostate cancer patients after radical prostatectomy, performed by treating physicians and/or patients.

While tumor infiltration was significantly lower in the early sRT group, no other statistically significant or clinically meaningful differences, such as positive surgical margins (25% vs. 19%), Gleason score 8–10 (21% vs. 31%), or pN1 status (8.3% vs. 6.9; all *p* ≥ 0.08), were identified. Among patients with high-risk features in both subgroups, the only significant difference was observed in the PSA level prior to radical prostatectomy in the early sRT group. All of the above findings indicates that all sRT groups were comparable regarding patient and tumor characteristics for MFS outcome, minimizing the risk for confounding or selection biases. However, Tilki et al., relying on *n* = 25.551 sRT patients, described significant predictors for prostate cancer-specific mortality such as age, pT3/4 stage, and Gleason score [15]. This discrepancy to our findings (*n* = 190) may stem from differences in cohort characteristics, sample size, follow-up durations, and study endpoints. Other literature has also identified these parameters such as the Gleason score and positive surgical margins as significant factors influencing influence outcomes [17–20].

Secondly, we made important observations regarding cancer-control outcomes for sRT patients. Specifically, MFS was significantly better in the early sRT group vs. sRT at PSA level > 0.5 ng/ml, with an MFS rate of 99% after 48 months compared to 80.4% in the standard sRT group. This resulted in an 8.4-fold higher risk of metastasis. All of the above findings about early sRT initiation at PSA level < 0.5 ng/ml continued to be independently predictive for MFS in further multivariable adjusted Cox regression models, adjusting for differences in baseline patient and tumor characteristics. These findings demonstrate the oncological need of early sRT initiation in BCR patients in clinical practice and are in line with previous findings. For example, Tilki et al. conducted a multicenter study with 1,832 patients comparing sRT versus no sRT in cases of BCR after radical prostatectomy at a median PSA level of 0.30 ng/ml. Their findings demonstrated a significantly higher MFS and overall survival (OS) in patients receiving sRT [6]. Similarly, a study by Trock et al. evaluated 635 patients receiving sRT at median PSA of 0.70 ng/ml, sRT patients with ADT at median PSA 0.90 ng/ml and patients with no sRT in case of BCR, assessing prostate cancer-specific survival rates, finding a significantly improved survival rate in patients undergoing sRT vs. no sRT [8]. However, the addition of ADT did not provide any further survival benefit in overall survival [8, 16, 21]. A review of ten studies, including 1212 patients treated between 2007 and 2016, confirmed improved BCR-free survival rates of sRT at a PSA level of ≤ 0.5 ng/ml vs. > 0.5 ng/ml [22]. In addition, Stephenson et al. analyzed the progression-free probability after sRT at different PSA levels. Their results demonstrated the highest progression-free probability when sRT was initiated at PSA levels ≤ 0.5 ng/ml [18]. Moreover, a recently published study by Lee et al. in 2023 reported significantly higher MFS of the patients receiving sRT at the PSA level of ≤ 0.5 ng/ml compared to those treated at PSA levels > 0.5 ng/ml [23]. Our findings align with aforementioned studies that recommend initiating sRT at PSA levels ≤ 0.5 ng/ml [6, 8, 18, 22, 23].

Compared to our findings, Tilki et al. analyzed a larger dataset with a lower cut-off (sRT initiation at PSA level > 0.25 ng/ml) focusing on patients from the University Hospital Hamburg-Eppendorf and the University of California. More specifically, the study analyzed 25,551 patients with pT2-4N0 or NxM0 prostate cancer who had undergone radical prostatectomy, with the primary endpoint of all-cause mortality. Within a six-year follow-up, Tilki et al. reported a significant reduction in all-cause mortality after ten years in the sRT group ≤ 0.25 ng/ml compared to patients receiving sRT at a PSA level > 0.25 ng/ml (9.3% vs. 14.5%; *p* = 0.008) [15]. As we have only limited data for this early cut-off, we could only apply this cut-off in our analysis as an exploratory analysis. Nevertheless, we could also prove qualitatively comparable results to the cut-off of 0.5 ng/ml.

Our study has several limitations. Firstly, it is a retrospective, single-center analysis with a sample size of 190 patients, may impact finding statistically significant differences. Further, these limitations may limit the generalizability of our findings to broader populations. Secondly, mortality outcomes may be influenced by confounding factors related to radiotherapy itself. Thereby, previous studies have reported a significantly higher mortality rate associated with radiotherapy, which could affect long-term survival outcomes beyond MFS [24, 25]. Furthermore, most patients in the early sRT group today undergo PSMA–positron emission tomography (PSMA-PET) for the evaluation of BCR. In this context, the initiation of sRT is often debated even in cases of negative PSMA-PET findings. Regarding the detection of metastases, PSMA-PET/CT was not applied in a standardized way during the evaluation of biochemical recurrence in our cohort. It is important to note that there is no available information on whether concomitant androgen deprivation therapy (ADT) was administered alongside sRT in our study population. Moreover, our data do not contain any information regarding the radiation fields or the administered radiation dose in the context of salvage radiotherapy. Our results support initiating sRT despite a negative PSMA-PET scan. This recommendation aligns with a study in 2021 analyzing the impact of PSMA-PET prior to sRT in BCR after radical prostatectomy [26].

Taken together, our real-world evidence study confirms the evidence of early sRT initiation at a PSA level < 0.5 ng/ml and earlier in terms of better MFS outcomes. This observation was also confirmed in multivariable adjusted analyses, providing robust and independent MFS outcomes for prostate cancer patients. Future studies should investigate the potential benefits of very-early sRT in a multicenter setting with larger patient cohorts and longer follow-up periods.

## Supplementary Information

Supplementary Material 1

Supplementary Material 2

## Data Availability

No datasets were generated or analysed during the current study.
