# Supplementary material for: Influence of PSA level at salvage radiotherapy on metastasis-free survival following radical prostatectomy
Source: World J Urol. 2025 Nov 21;43(1):713. doi: 10.1007/s00345-025-05840-w (PMC12638372; doi:10.1007/s00345-025-05840-w)
Supplement: Supplementary file 1 — Supplementary Material 1 [file 345_2025_5840_MOESM1_ESM.docx]

Supplemental Table 3: Univariable und multivariable Cox regression models predicting metatasis-free survival

|  | **Univariable** | | | **Multivariable** | | |
| --- | --- | --- | --- | --- | --- | --- |
| **MFS** | **HR** | **CI** | **p-value** | **HR** | **CI** | **p-value** |
| sRT PSA <0.5 ng/ml | 8.4376 | 1.6948-42.0065 | **0.0092** | 8.2363 | 1.4131-48.006 | **0.0191** |
| Age at sRT | 0.9641 | 0.8712-1.067 | 0.4797 | 0.92 | 0.8084-1.0471 | 0.2066 |
| pT3-4 | 2.6463 | 0.5333-13.1312 | 0.2338 | 2.5835 | 0.4187-15.9418 | 0.3066 |
| pN1 | 1.3261 | 0.162-10.8594 | 0.7925 | 0 | 0-Inf | 0.999 |
| R1 | 0.7388 | 0.0902-6.0542 | 0.7778 | 1.0136 | 0.1086-9.4646 | 0.9905 |
| Highest PSA | 0.9946 | 0.9408-1.0515 | 0.8486 | 0.9818 | 0.9056-1.0643 | 0.6551 |
| Gleason 8-10 | 0.516 | 0.0633-4.2037 | 0.5364 | 0.6526 | 0.0674-6.3223 | 0.7126 |
| ECOG 1-2 | 0 | 0-Inf | 0.9982 | 0 | 0-Inf | 0.9994 |

CI = Confidence interval; ECOG = Eastern Cooperative Oncology Group; HR = Hazard Ratio; MFS = Metastasis-free survival; sRT = salvage radiotherapy; PSA = Prostate specific antigene.
