# Supplementary material for: Influence of PSA level at salvage radiotherapy on metastasis-free survival following radical prostatectomy
Source: World J Urol. 2025 Nov 21;43(1):713. doi: 10.1007/s00345-025-05840-w (PMC12638372; doi:10.1007/s00345-025-05840-w)
Supplement: Supplementary file 2 — Supplementary Material 2 [file 345_2025_5840_MOESM2_ESM.docx]

Supplemental Table 4: Univariable und multivariable Cox regression models predicting metatasis-free survival at patients with high-risk features

|  | **Univariable** | | | **Multivariable** | | |
| --- | --- | --- | --- | --- | --- | --- |
| **MFS** | **HR** | **CI** | **p-value** | **HR** | **CI** | **p-value** |
| sRT PSA <0.5 ng/ml | 12.6899 | 1.4716-109.4295 | **0.0208** | 55.6463 | 1.507-2054.8163 | **0.0291** |
| Age at sRT | 0.9354 | 0.8396-1.0421 | 0.2254 | 0.7947 | 0.6331-0.9975 | 0.0475 |
| pT3-4 | 77854944.0435 | 0-Inf | 0.9987 | 809772197.2683 | 0-Inf | 0.9993 |
| pN1 | 1.0123 | 0.117-8.758 | 0.9911 | 0 | 0-Inf | 0.9994 |
| R1 | 0.8247 | 0.0951-7.151 | 0.8611 | 6.5849 | 0.205-211.5253 | 0.287 |
| Highest PSA | 0.9922 | 0.9308-1.0578 | 0.8116 | 0.9481 | 0.8258-1.0885 | 0.4494 |
| Gleason 8-10 | 0.3538 | 0.0413-3.0343 | 0.3433 | 1.1812 | 0.0641-21.7617 | 0.9108 |
| ECOG 1-2 | 0 | 0-Inf | 0.9988 | 0 | 0-Inf | 0.9997 |

CI = Confidence interval; ECOG = Eastern Cooperative Oncology Group; HR = Hazard Ratio; MFS = Metastasis-free survival; sRT = salvage radiotherapy; PSA = Prostate specific antigene.
